# Impact of a single session of hyperbaric oxygen therapy on the healthy retina

**DOI:** 10.3389/fopht.2025.1652282

**Published:** 2025-10-20

**Authors:** Nur Demir, Selin Gamze Sumen, Belma Kayhan

**Affiliations:** 1 Ophthalmology Department, Sultan 2. Abdulhamid Han Training and Research Hospital, University of Health Sciences, Istanbul, Türkiye; 2 Hyperbaric and Undersea Medicine Department, Dr. Lutfi Kirdar Kartal Training and Research Hospital, Istanbul, Türkiye

**Keywords:** choroidal thickness, full-field electroretinography, hyperbaric oxygen therapy, optical coherence tomography, photoreceptors, retina, retina pigment epithelium

## Abstract

**Background:**

Hyperoxia induced by hyperbaric oxygen therapy (HBOT) may lead to retinal vasoconstriction and the generation of reactive oxygen species. This study aims to investigate the effects of a single session of HBOT on the healthy retina using full-field electroretinography (ffERG) and spectral-domain optical coherence tomography (SD-OCT).

**Methods:**

Twenty patients diagnosed with either sensorineural hearing loss or avascular necrosis, all of whom had an indication for HBOT, were included in the study. A comprehensive ophthalmologic examination, along with ffERG and SD-OCT assessments of the retinal layers and choroid, were performed both before and within 24 hours after the first HBOT session.

**Results:**

The mean age of the participants was 43.2 ± 11.4 years (range, 18–66 years). A statistically significant difference was observed only in the scotopic 0.01 ERG b-wave amplitude before and after HBOT (p = 0.029). The retinal pigment epithelium in the 3-mm nasal subfield of the Early Treatment Diabetic Retinopathy Study (ETDRS) grid demonstrated a statistically significant thickening after the first HBOT session (p = 0.023).

**Conclusion:**

A single session of HBOT induced an acute alteration in rod–bipolar cell function, as evidenced by impaired electrophysiological responses. Additional studies are necessary to clarify the duration and potential reversibility of the observed electrophysiological impairment.

## Introduction

Hyperbaric oxygen therapy (HBOT) involves the administration of 100% oxygen (O_2_) to patients within a closed environment subjected to pressures exceeding atmospheric levels. This therapeutic approach, in which O_2_ is employed as a medication, is recognized for its efficacy in treating various diseases, as endorsed by international scientific authorities (1). The primary mechanisms by which HBOT exerts its effects are hyperoxygenation and the reduction of gas bubble size (1, 2). Hyperoxygenation enhances both plasma and tissue oxygen concentration by increasing the solubility of oxygen under high pressure, as articulated by Henry’s law (2). According to Boyle’s law, an increase in pressure corresponds to a decrease in the volume of gas (1, 2). Furthermore, HBOT reduces tissue edema and promotes wound healing due to its vasoconstrictive, angiogenic, and anti-inflammatory properties (1, 2).

HBOT is frequently employed to treat several medical conditions, including decompression sickness, arterial gas embolism, carbon monoxide poisoning, burns, acute idiopathic sensorineural hearing loss, and acute blindness resulting from central retinal artery occlusion (CRAO), in addition to chronic osteomyelitis, among others (2). HBOT appears to have no significant impact on choroidal blood flow or vascular volume (3). However, it enhances oxygen concentration within the inner retina via choroidal oxygen diffusion, even in the presence of CRAO (4). The elevation of blood and tissue O_2_ levels consequently leads to an increase in reactive O_2_ and nitrogen species (5). Benedetti et al. investigated the effects of HBOT on plasma reactive oxygen species (ROS), demonstrating that ROS levels rise from the initial HBOT session and stabilize at moderate levels after approximately fifteen sessions (5).

William and Bridge examined the electrophysiological effects of HBOT in animal models. Their research indicated that the b-wave is more susceptible to HBOT than the a-wave, and they noted that alterations in the b-wave did not consistently revert to baseline (6). Currently, there is only one study addressing the electrophysiological effect of HBOT on healthy human eyes. This study demonstrated impairment in the amplitudes of both the a- and b-waves, as measured by full-field electroretinography (ffERG), within 24 hours following the 10th session of HBOT (7). The objective of the present study was to investigate whether electrophysiological changes occur as early as the first session of HBOT. The ffERG and spectral-domain optical coherence tomography (SD-OCT) were employed to assess both electrophysiological and structural changes within the retina.

## Methods

This prospective study was approved by the medical ethics committee of the hospital. It was conducted in accordance with the principles of the Declaration of Helsinki. Written informed consent was obtained from all participants.

Patients without any ocular diseases who required HBOT due to diagnoses of sensorineural hearing loss (SNHL) or avascular necrosis were included in the study. Among the SNHL group, oral or intratympanic steroid therapy was administered prior to HBOT. Importantly, comprehensive ophthalmologic examinations confirmed that none of these patients, nor any of the other participants, exhibited ocular pathology attributable to these treatments. Prior to HBOT, all participants underwent a detailed ophthalmologic evaluation, which revealed no ocular conditions that could influence the study outcomes.

The exclusion criteria included glaucoma, spherical or cylindrical corrections exceeding ± 3.0 diopters, a history of ocular trauma, a history of intraocular surgery, individuals under the age of 18, previous administration of HBOT, any presence of retinal and choroidal diseases, diabetes mellitus, nephrological, inflammatory, endocrine, or respiratory diseases, as well as any other cardiovascular conditions.

A comprehensive ophthalmologic examination, consisting of best corrected visual acuity (BCVA), slit-lamp biomicroscopy, intraocular pressure (IOP) measurement, and tropicamide-dilated fundus examination, was performed both before and within 18–24 hours after the first session of HBOT. Furthermore, ffERG and SD-OCT measurements of the retinal layers and choroid were conducted before and after the first session of HBOT within the same 18–24-hour timeframe.

The measurement of retinal layers was performed using spectral-domain optical coherence tomography (Version 1.10.4.0, Software_V6.16.2, Heidelberg Engineering, Heidelberg, Germany), preceded by pupil dilation. The scan encompassed a 30×20 degree cube with 25 raster lines, each separated by 240 μm. The analysis included total retinal thickness as well as the thicknesses of the retinal nerve fiber layer (RNFL), ganglion cell layer (GCL), inner plexiform layer (IPL), inner nuclear layer (INL), outer plexiform layer (OPL), outer nuclear layer (ONL), and retinal pigment epithelium (RPE) within the central 1-mm and 3-mm subfields of the Early Treatment Diabetic Retinopathy Study (ETDRS) grid.

Subfoveal, nasal and temporal choroidal thickness (CT) were measured 1000 μm distant from the fovea with SD-OCT. All measurements were performed by the same technician. Retinal layers segmentation was performed automatically but segmentation errors were corrected manually.

Patients received HBOT at a pressure of 243 kPa in routine sessions conducted within a multiplace chamber accommodating twelve individuals (Zyron 12 Multiplace MLT 09, Hipertech). The treatment protocol consisted of 90 minutes of O_2_ exposure, delivered in three 30-minute intervals interspersed with 5-minute air breaks. During therapy, patients inhaled 100% O_2_ via a mask.

Full-field electroretinography was performed using the RETI-port system (Roland Consult, Brandenburg, Germany) in accordance with the standards of the International Society for Clinical Electrophysiology of Vision (ISCEV). All recordings were conducted by a single trained technician to ensure consistency. Prior to testing, pupils were pharmacologically dilated. Topical anesthesia was applied, and electrodes were positioned appropriately. After 20 minutes of dark adaptation, dark-adapted (DA) electroretinograms were recorded, followed by 10 minutes of light adaptation prior to the acquisition of light-adapted (LA) electroretinograms. The standard ISCEV ffERG protocol includes the following five tests:

1. DA 0.01 ERG – Response of rod and bipolar cell activity to 0.01 cd.s/m^2^.
2. DA 3.0 ERG – Represents combined responses from rod and cone systems, including bipolar cells, to 3.0 cd.s/m^2^.
3. DA 10.0 ERG – Similar to DA 3.0, but with larger a-waves for enhanced analysis of rod and cone responses to 10.0 cd.s/m^2^.
4. LA 3.0 ERG – Measures cone system function response to 3.0 cd.s/m^2^; the a-wave originates from cone photoreceptors and OFF-cone bipolar cells, while the b-wave is generated by both ON and OFF cone bipolar cells.
5. LA 30-Hz Flicker ERG – Represents cone response to 3.0 cd.s/m^2^ at 30 Hz.

The same ophthalmologist examined all patients and recorded data.

### Statistics

The IBM SPSS version 26.0 software was utilized in the statistical analysis of this study. The recorded ffERG and SD-OCT values before and after the first session of HBOT were compared. Descriptive statistics were computed, including the median, minimum-maximum values, and mean ± standard deviation. The Shapiro-Wilk test was employed to assess the distribution of parameters. The paired samples t-test was applied to compare normally distributed values, while the Wilcoxon signed-rank test was utilized to compare data that did not follow a normal distribution. All statistical analyses were conducted under a 95% confidence interval, with a significance level at p<0.05.

## Results

A total of 40 eyes from 20 patients were evaluated in this study. HBOT was administered to 5 patients with a diagnosis of SNHL and to 15 patients with a diagnosis of avascular necrosis. Six participants (30%) were female, and 14 (70%) were male. The mean age of the participants was 43.2 ± 11.4 years (range, 18–66 years).

BCVA was 20/20 in all examined eyes. The median IOP was 16 mmHg (range, 13.5–22 mmHg) before HBOT and 15.25 mmHg (range, 13.5–22 mmHg) after the initial HBOT session. This difference was not statistically significant (p = 0.528). The mean spherical equivalent was 0.09 ± 0.4 D (median, 0 D) before HBOT and 0.06 ± 0.4 D (median, 0 D) after HBOT.

The ffERG examination was conducted on forty eyes. SD-OCT measurements were absent for three patients; therefore, SD-OCT values for thirty-four eyes were analyzed. Among these, four eyes presented with small or medium-sized drusen.

A statistically significant difference was observed only in the scotopic 0.01 ERG b-wave amplitude values of the participants before and after HBOT (p = 0.029, Wilcoxon signed-rank test; **Table 1**). In contrast, no significant changes were detected in the other ffERG parameters following the initial HBOT session (**Table 1**).

**Table 1:** The full-field electroretinography responses before and after the first session of hyperbaric oxygen treatment.

	Before HBOT		After HBOT		
Full-field ERG	Median	Mean ± SD	Median	Mean ± SD	P
0.01 ERG IT	92.55	91.50 ± 6.30	92.25	89.60 ± 7.60	0.366¹
Scotopic 0.01 ERG b Amplitude	145.50	174.20 ± 91.30	139.00	146.40 ± 39.30	0.029*²
Scotopic 3.0 ERG IT a	22.37	22.10 ± 1.70	22.38	21.10 ± 3.20	0.190²
Scotopic 3.0 ERG IT b	47.32	46.80 ± 2.80	47.10	47.20 ± 2.40	0.722²
Scotopic 3.0 a Amplitude	175.00	181.50 ± 35.50	170.50	184.70 ± 46.30	0.519¹
Scotopic 3.0 b Amplitude	333.00	324.80 ± 64.10	294.00	312.20 ± 73.10	0.341¹
Scotopic 10 IT a	16.32	16.80 ± 1.70	16.50	17.20 ± 2.20	0.452²
Scotopic 10 IT b	49.60	47.40 ± 3.40	49.45	47.90 ± 3.00	0.305²
Scotopic 10 a Amplitude	240.75	241.40 ± 53.00	213.75	232.50 ± 54.70	0.405¹
Scotopic 10 b Amplitude	347.00	348.90 ± 76.60	323.75	337.30 ± 74.10	0.455¹
Scotopic 3.0 N2	21.32	21.20 ± 0.90	21.55	21.30 ± 1.30	0.709¹
Scotopic 3.0 P2	25.65	25.70 ± 0.70	25.87	25.70 ± 0.90	0.589²
Scotopic 3.0 OS2	62.02	62.50 ± 21.40	60.47	65.50 ± 26.70	0.423¹
Photopic 3.0 IT a	15.37	15.30 ± 0.80	15.45	15.20 ± 1.10	0.526¹
Photopic 3.0 IT b	30.50	30.70 ± 1.10	30.72	30.80 ± 10.00	0.390¹
Photopic 3.0 a Amplitude	36.12	36.00 ± 10.00	33.45	36.80 ± 13.70	0.675¹
Photopic 3.0 b Amplitude	150.50	152.70 ± 39.20	143.00	156.80 ± 47.40	0.464¹
Photopic 30-Hz flicker IT P1	17.90	18.20 ± 0.60	17.90	18.00 ± 0.30	0.362²
Photopic 30-Hz flicker N1-P1	26.85	26.60 ± 0.50	26.77	26.60 ± 0.50	0.418²
Photopic 30-Hz flicker IT N1	85.22	87.20 ± 20.20	87.62	94.50 ± 25.70	0.127²

Paired samples t test^1^; Wilcoxon signed ranks test^2^*. P < 0.05 indicates statistical significance.

Full-field ERG, full-field electroretinography; HBOT, hyperbaric oxygen treatment; IT, implicit time; SD, standard deviation.

Analysis of the RPE thickness revealed a statistically significant increase in the 3-mm nasal subfield of the ETDRS grid after HBOT compared with the pre-treatment values (p = 0.023, paired-samples t-test; **Table 2**). CT showed no significant change in the subfoveal, nasal, or temporal measurements (**Table 2**).

**Table 2:** A comparative analysis of total retinal thickness and individual retinal layer thicknesses before and after a single session of hyperbaric oxygen therapy.

	Before HBOT		After HBOT		
ETDRS Ring	Median	Mean ± SD	Median	Mean ± SD	P
RT					
Central 1-mm	254.00	260.24 ± 19.20	254.50	259.50 ± 20.10	0.449¹
Superior 3-mm	347.50	344.94 ± 15.10	345.00	346.00 ± 14.30	0.310¹
Inferior 3-mm	343.50	344.32 ± 14.30	341.00	343.76 ± 13.70	0.548¹
Nasal 3-mm	339.00	341.88 ± 21.70	343.00	345.26 ± 16.60	0.296¹
Temporal 3-mm	332.50	331.03 ± 13.50	333.00	332.09 ± 15.10	0.226¹
RNFL					
Central 1-mm	10.50	11.09 ± 2.20	10.50	10.71 ± 2.50	0.154¹
Superior 3-mm	23.00	23.32 ± 2.40	23.00	23.47 ± 2.70	0.770¹
Inferior 3-mm	26.00	25.91 ± 2.40	26.00	26.00 ± 3.10	0.843¹
Nasal 3-mm	21.00	20.82 ± 1.90	21.00	21.00 ± 2.00	0.638²
Temporal 3-mm	17.50	17.71 ± 0.80	17.50	17.38 ± 1.00	0.160²
GCL					
Central 1-mm	12.00	12.53 ± 3.40	12.00	12.24 ± 3.30	0.532¹
Superior 3-mm	53.50	51.94 ± 5.90	53.50	52.35 ± 5.40	0.414¹
Inferior 3-mm	53.50	52.85 ± 4.80	52.50	52.71 ± 5.10	0.693¹
Nasal 3-mm	53.50	50.82 ± 6.90	50.00	50.50 ± 6.70	0.658²
Temporal 3-mm	47.00	46.82 ± 5.70	48.00	47.21 ± 5.70	0.453¹
IPL					
Central 1-mm	18.00	18.21 ± 2.90	18.00	17.82 ± 3.50	0.307¹
Superior 3-mm	42.00	41.18 ± 3.80	42.50	41.41 ± 3.40	0.652²
Inferior 3-mm	42.00	41.59 ± 3.10	41.50	41.24 ± 3.30	0.210¹
Nasal 3-mm	42.00	42.03 ± 4.30	42.00	42.12 ± 3.90	0.787¹
Temporal 3-mm	42.50	41.26 ± 3.50	42.00	41.26 ± 3.30	1.000¹
INL					
Central 1-mm	17.00	17.12 ± 4.00	17.00	16.59 ± 3.40	0.409¹
Superior 3-mm	42.00	42.74 ± 3.50	43.00	42.35 ± 2.00	0.660²
Inferior 3-mm	42.00	42.24 ± 2.40	42.00	42.15 ± 2.60	0.813¹
Nasal 3-mm	42.00	41.65 ± 2.80	41.50	42.09 ± 3.10	0.577¹
Temporal 3-mm	39.50	39.41 ± 2.40	38.50	38.94 ± 2.00	0.436¹
OPL					
Central 1-mm	22.00	21.26 ± 3.60	20.50	22.03 ± 4.30	0.367¹
Superior 3-mm	32.50	35.94 ± 8.20	32.50	35.88 ± 7.80	0.831²
Inferior 3-mm	29.00	30.24 ± 4.30	29.00	30.97 ± 5.20	0.815²
Nasal 3-mm	33.00	33.00 ± 5.10	30.50	33.35 ± 7.80	0.981²
Temporal 3-mm	31.00	31.29 ± 3.70	32.00	31.47 ± 2.80	0.587²
ONL					
Central 1-mm	95.00	93.65 ± 10.90	95.00	93.47 ± 10.10	0.891¹
Superior 3-mm	69.00	67.79 ± 13.10	65.50	68.35 ± 11.40	0.756¹
Inferior 3-mm	69.00	71.26 ± 7.60	71.00	70.21 ± 8.30	0.168¹
Nasal 3-mm	75.00	74.32 ± 10.40	74.50	73.09 ± 8.90	0.523¹
Temporal 3-mm	73.50	73.18 ± 8.80	74.00	73.29 ± 8.00	0.914¹
RPE					
Central 1-mm	16.50	16.41 ± 1.90	16.50	16.32 ± 1.60	0.794¹
Superior 3-mm	16.00	15.53 ± 1.50	15.50	15.76 ± 1.80	0.375¹
Inferior 3-mm	15.00	14.91 ± 1.20	15.00	15.00 ± 1.70	0.756¹
Nasal 3-mm	15.50	15.38 ± 1.50	16.50	15.82 ± 1.60	0.023*¹
Temporal 3-mm	14.50	14.50 ± 1.20	14.50	14.68 ± 1.40	0.393¹
CT					
Nasal 1-mm	345.75	350.36 ± 56.10	361.00	362.46 ± 70.00	0.415¹
Central	346.75	349.89 ± 45.30	355.50	356.69 ± 46.10	0.203¹
Temporal 1-mm	356.00	346.43 ± 84.00	317.50	339.96 ± 89.40	0.764¹

Paired samples t test^1^; Wilcoxon signed ranks test^2^*. P < 0.05 indicates statistical significance.

ETDRS grid, Early Treatment Diabetic Retinopathy Study grid; RT, full retinal thickness; RNFL, retinal nerve fiber layer; GCL, ganglion cell layer; IPL, inner plexiform layer; INL, inner nuclear layer; OPL, outer plexiform layer; ONL, outer nuclear layer; RPE, retina pigment epithelial layer; SD, standard deviation.

## Discussion

In this study, we assessed the acute effects of HBOT on the healthy retina following the initial treatment. The investigation revealed a reduction in the b-wave amplitude of scotopic 0.01 ffERG in the healthy retina within 24 hours after the first session of HBOT.

Inhaling 100% O_2_ at 1 atmosphere absolute (ATA) elevates arterial partial pressure of oxygen (PaO_2_) from 91.7 ± 6.8 mmHg to 576.7 ± 18.9 mmHg (8). This increase in blood O_2_ levels diminishes retinal blood flow by up to 50% (9), while choroidal blood flow remains unaffected (3). The deep capillary plexus (DCP) exhibit vasoconstriction to protect photoreceptors during 100% O_2_ inhalation (10). Systemic hyperoxia leads to a reduction of both retinal arterial and venous diameters, thereby decreasing oxygen delivery to the eye by 50-70% (9). However, the reduction in oxygen supply to the retina can be compensated by transporting choroidal O_2_ to the inner retina, which ascends to 228 ± 21 mmHg during O_2_ inhalation (9, 11).

HBOT elevates blood and tissue oxygen levels beyond that achieved by inhaling 100% O_2_ at 1.0 ATA. Under a pressure of 3.0 ATA, it can increase PaO_2_ to as high as 2000 mmHg (12). Çevik et al. reported a reduction in vessel diameter in both the superior capillary plexus and the DCP (13). The choroid enhances the PaO_2_ of the outer retinal layers to significantly high levels through the process of oxygen diffusion, subsequently inducing oxidative stress (11). This oxidative stress predominantly affects photoreceptors, potentially leading to apoptosis and the consequent loss of these cells (14).

To date, no study has evaluated the electrophysiological response to a single session of HBOT in the healthy human eye. An animal study demonstrated that exposure to 100% O_2_ at 4 ATA resulted in retinal edema, retinal folding, and complete obliteration of the electroretinographic response (15). In a previously published investigation by our research team, the ffERG response was assessed in healthy eyes following ten HBOT sessions, within 24 hours of the final session. In that study, significant reductions were observed in scotopic b-wave amplitude, scotopic 10 a-wave amplitude, photopic 3.0 a-wave amplitude, and photopic 30 Hz flicker ERG N1–P1 amplitude, leading to the conclusion that the photoreceptor layer was affected (7).

In the present study, the scotopic 0.01 ERG b-wave amplitude exhibited a significant decrease following the initial session of HBOT. The scotopic 0.01 b-wave reflects the activity of rod-bipolar cells (16). The study conducted by Bhatt et al. concluded that oxidative stress elevates ROS levels in rods and cones (17). A study involving rabbits indicated that exposure to 2.5 ATA of HBO decreased b-wave amplitude approximately 2.5 hours later. Notably, the a-wave amplitude also diminished, but at higher pressures. The b-wave was determined to be more susceptible to hyperoxia compared to the a-wave (6). Moreover, even exposure to 1 ATA of O_2_ reduced b-wave amplitude after 25–36 hours in another animal study (18).

Following HBOT, RPE thickness increased across all regions of the 3 mm ETDRS grid in this study; however, the increase reached statistical significance only in the nasal region. The acute effect of HBOT after the first session was evaluated by Sayın et al. using SD-OCT, but no changes in retinal layer thicknesses were observed (19). Although both the present investigation and the study by Sayın et al. assessed the immediate effects of a single HBOT session, the protocols employed differed. Sayın et al. applied a treatment duration of 120 minutes at a pressure of 2.4 ATA, whereas in the present study HBOT was administered as a 90-minute session with 100% O_2_ at the same pressure. An additional 30 minutes was allocated for air breaks and for regulating the pressure within the hyperbaric chamber. Central macular thickness was found to be decreased within 30 minutes after HBOT in another study (20). Furthermore, Nachman-Clewner et al. reported that a 5-week exposure to HBO resulted in central photoreceptor loss without observable alterations in the RPE (14). Considering previous studies, the RPE thickening observed in the present study may be regarded as an incidental finding. If we consider this finding to be accurate and non-coincidental, two probable mechanisms may be proposed to explain the observed phenomenon.

The first plausible mechanism proposes that acute oxidative damage to the RPE may occur. Elevated tissue O_2_ pressure, which can reach up to 400 mmHg, induces the formation of ROS during HBOT (21). This hyperoxic oxidative stress predominantly affects photoreceptors as well as the RPE. In a murine model, three weeks of HBO exposure led to a loss of photoreceptors, thinning of the outer nuclear layer, swelling of the inner segment junction, and darkening of the outer segment junction (14).

The other probable mechanism is that the increased permeability of the blood-retina barrier (BRB) may contribute to the thickening of the RPE. The existing literature has primarily evaluated the effects of HBOT on conditions such as diabetic retinopathy, retinal vein occlusion, and arterial occlusion. In rats with diabetic retinopathy, BRB permeability improved after 3 months of HBO, as demonstrated by Chang et al. (22). Furthermore, HBO has been shown to alleviate disruption of the blood-brain barrier (BBB) in various animal models of ischemic brain lesions (23, 24). However, Çevik et al. demonstrated that, unlike in ischemic lesions, HBO induced impairment of the BBB in healthy rats (25). Notably, no research has been published regarding the effects of HBO on the healthy BRB. Tight junctions, which are present in both the BRB and BBB, represent a common structural barrier (26). These observations raise the possibility that HBOT exerts differential effects on the BRB in ischemic versus healthy retinal tissues.

HBOT has been shown to reduce ROS and pro-oxidant enzyme levels in ischemic lesions; in contrast, it increases ROS in healthy tissue (27). In an animal study, HBO-induced oxidative stress either killed healthy rats or conferred resistance to lethal oxidative stress by activating signaling and adaptive processes (28). Similarly, preconditioning RPE cells with low doses of H_2_O_2_ reduced the cytotoxic effects of a subsequent lethal dose of H_2_O_2_ in another study (29). When the above studies are considered alongside the outcomes of the present investigation the following questions raises: whether the effects of HBOT differ between diseased and healthy retinas, and whether retinal ischemia confers a preconditioning effect that mitigates HBOT-related hyperoxic stress. These questions warrant investigation in future studies.

In conclusion, this study demonstrates that a single session of HBOT has an effect on the b-wave in ffERG, indicating an impact on rod-bipolar cell functionality. Additional research is warranted to determine whether the observed adverse effect on rod-bipolar function is transient or persistent. In the event that electrophysiological parameters do not return to baseline, modifications to HBOT protocols may be necessary.

## Data Availability

The original contributions presented in the study are included in the article/supplementary material, further inquiries can be directed to the corresponding author.
